# Unpacking the p-factor. Associations Between Maladaptive Personality Traits and General Psychopathology in Female and Male Adolescents

**DOI:** 10.1007/s10802-023-01146-w

**Published:** 2023-11-08

**Authors:** Ilaria Maria Antonietta Benzi, Andrea Fontana, Rossella Di Pierro, Laura Parolin, Karin Ensink

**Affiliations:** 1 Department of Brain and Behavioral Sciences, University of Pavia, Piazza Botta Adorno Antoniotto, 11, 27100, Pavia, Italy; 2 Department of Human Science, LUMSA University, Rome, Italy; 3 Department of Psychology, University of Milano-Bicocca, Milan, Italy; 4 Department of Psychology, Laval University, Quebec, QC Canada

**Keywords:** General psychopathology, p-factor, Adolescence, Borderline traits, Narcissistic traits, Self-development, Self-dysregulation, Personality pathology

## Abstract

Adolescence is a period of rapid physical, psychological, and neural maturation that makes youth vulnerable to emerging psychopathology, highlighting the need for improved identification of psychopathology risk indicators. Recently, a higher-order latent psychopathology factor (p-factor) was identified that explains latent liability for psychopathology beyond internalizing and externalizing difficulties. However, recent proposals suggest reconceptualizing the p-factor model in terms of impairments in personality encompassing difficulties in both self-regulation (borderline features) and self-esteem (narcissistic features), but this remains untested. To address this, this study examined the p-factor structure and the contribution of borderline and narcissistic features using two cross-sectional data collections. In Study 1, 974 cisgender adolescents (63% assigned females at birth; age range: 13–19; M*age* = 16.68, *SD* = 1.40) reported on internalizing and externalizing problems (YSR) to test via structural equation models (SEM) different theoretical models for adolescent psychopathology. In Study 2, 725 cisgender adolescents (64.5% assigned females at birth; age range: 13–19; M*age* = 16.22, *SD* = 1.32) reported internalizing and externalizing problems (YSR), borderline personality features (BPFSC-11), and narcissistic personality traits (PNI), to explore, via SEM, the contribution of borderline and narcissistic traits to the p-factor and accounting for gender differences. Results confirmed the utility of a bi-factor model in adolescence. Furthermore, findings highlighted the contribution of borderline features and narcissistic vulnerability to general psychopathology. The study provides the first evidence supporting a p-factor model reconceptualized in terms of personality impairments encompassing difficulties in self-regulation and self-esteem in adolescents.

**Supplementary Information:**

The online version contains supplementary material available at 10.1007/s10802-023-01146-w.

## Introduction

### A General Factor for Psychopathology in Adolescence

Adolescence is a critical developmental period of rapid physical, psychological, and neural maturation (Casey et al., 2008), coinciding with increased risk-taking and engaging with challenges inherent to the transition to adulthood. These challenges include consolidating a stable, coherent sense of self, developing relationships with peers and establishing early sexual/romantic relationships, as well as taking on independent adult role functions (Ensink et al., 2015; Erikson, 1959; Kernberg et al., 2000). These factors make youth vulnerable to emerging psychopathology, highlighting the need for improved identification of psychopathology risk indicators. During adolescence, impairments in self and relational functioning may increase vulnerability to the onset of frank psychopathology or to sub-threshold conditions that anticipate the exacerbation of psychopathology in emerging adulthood (Merikangas et al., 2022). For these reasons, it is essential to understand dispositional vulnerability to psychopathology in adolescence to improve early identification and intervention (Chanen et al., 2017; Crocetti et al., 2013; Hicks et al., 2007; Sharp & Wall, 2018).

Until recently, frameworks for understanding psychopathology risk have mainly been symptom-focused, with a categorical approach used to differentiate psychopathology. However, a growing body of research challenges the categorical approach, given high comorbidity rates and overlapping disorder presentations (Kessler et al., 2005). Furthermore, clinicians and researchers have proposed that dimensional approaches may be more appropriate for understanding psychopathology and informing treatment (Forbes et al., 2016; Wright et al., 2013). As a result, the major psychiatric diagnostic manuals have incorporated dimensional taxonomies (APA, 2013; Hopwood et al., 2018; Krueger & Bezdjian, 2009; Möller, 2009; Schmeck & Birkhölzer, 2021).

In this context, the 2014 seminal paper by Caspi and colleagues (2014) identified the psychopathology p-factor, a transdiagnostic psychopathology dimension superordinate to individual mental disorders. Like the G factor of intelligence, the psychopathology p-factor enables understanding of features shared across disorders. The psychopathology p-factor is a latent dimension that encapsulates individuals’ psychopathology across internalizing, externalizing, and thought disorders. Furthermore, the p-factor is hypothesized to tap into vulnerability for psychopathology and better predict the risk of developing psychopathology (Caspi et al., 2014). From a developmental perspective, Miller et al. (2019) confirmed the early emergence of the p-factor, suggesting its crucial role in understanding lifelong mental health trajectories. Studies with adolescent populations have replicated the structure proposed by Caspi and colleagues and demonstrated its longitudinal stability (Allegrini et al., 2020; Laceulle et al., 2015; Murray et al., 2016; Neumann et al., 2020; Patalay et al., 2015).

Since identifying the p-factor, a debate has evolved regarding its significance (Allegrini et al., 2020; Lahey et al., 2012; Murray et al., 2016; Ronald, 2019). On the one hand, some have suggested that it could be a mere statistical artifice (Bonifay et al., 2017) and nothing more than “the sum of its parts” (Fried et al., 2021); on the other, it offers the possibility of detecting a transdiagnostic factor helpful in explaining the shared variance across psychopathology presentations not captured by traditional models (Carver et al., 2017; Laceulle et al., 2020; Tackett et al., 2013). Furthermore, it allows for a clearer developmental understanding of the stability of psychopathology pathways and potentially enables the prediction of future psychopathology risk (Sharp & Wall, 2018).

### Association of Personality Pathology and General Psychopathology: Self-dysregulation as a Nexus

Revising Caspi’s original model, Sharp and Wall (2018) propose that the developmental trajectory of the p-factor should encompass personality pathology in addition to internalizing and externalizing difficulties and thought disorders. Maladaptive personality traits are associated with internalizing and externalizing dimensions of psychopathology (Gjerde et al., 2023; Sharp & Wall, 2018; Shields et al., 2021). Indeed, previous research on emerging personality pathology in adolescents suggests that there is an interplay between internalizing and externalizing features and borderline personality disorder features indicative of impairments in self-development (i.e., instability of self-image, emotional dysregulation and relationship problems) (Benzi et al., 2022, 2023a, b; Biberdzic et al., 2022; Bleiberg et al., 2012; Chanen et al., 2017; Conway et al., 2015, 2016; Stepp et al., 2016). Sharp and Wall (2018) suggest integrating Criterion A of the Alternative Model for Personality Disorders (AMPD) adopted by the DSM-5 (APA, 2013), which focuses on impairments in self and interpersonal functioning (Sharp & Wall, 2018; Sharp et al., 2018). This is likely particularly relevant in adolescents because, as Sharp points out, “*developmental research suggests that Criterion A concepts (identity, self-direction, empathy, and intimacy) coalesce around the development of self, marking a discontinuous (qualitative) shift in development that enables the adolescent to take on independent adult role function, which is demanded from the environment*” (Sharp, 2020, p. 202). Consistent with this, impaired self-development has been identified by studies showing that borderline personality disorder features are robust markers of personality pathology (Biberdzic et al., 2022; Sharp & Fonagy, 2015; Sharp & Wall, 2018). Gender differences in borderline features have been identified by some studies showing that males display more aggressive and antisocial traits (Bradley et al., 2005), while other studies find more commonalities than differences (Johnson et al., 2003; Silberschmidt et al., 2015).

Despite the need for an integrated framework of personality, personality pathology, and psychopathology in youth, research on the association of maladaptive personality traits and the p-factor in adolescents is scarce. Only one recent study explored the longitudinal associations between the p-factor and borderline personality features and found that p predicted within-person change at different ages (Choate et al., 2023). Studies that have examined personality and the p-factor have mainly focused on the Big Five model of personality traits (Carragher et al., 2016; Caspi et al., 2014; Castellanos-Ryan et al., 2016; Tackett et al., 2013) and found that adolescent psychopathology is associated with personality traits such as impulsivity and hopelessness (Carragher et al., 2016). Furthermore, high disinhibition/impulsivity, low agreeableness, high neuroticism, and hopelessness are also associated with general psychopathology (Castellanos-Ryan et al., 2016).

**Fig. 1:**
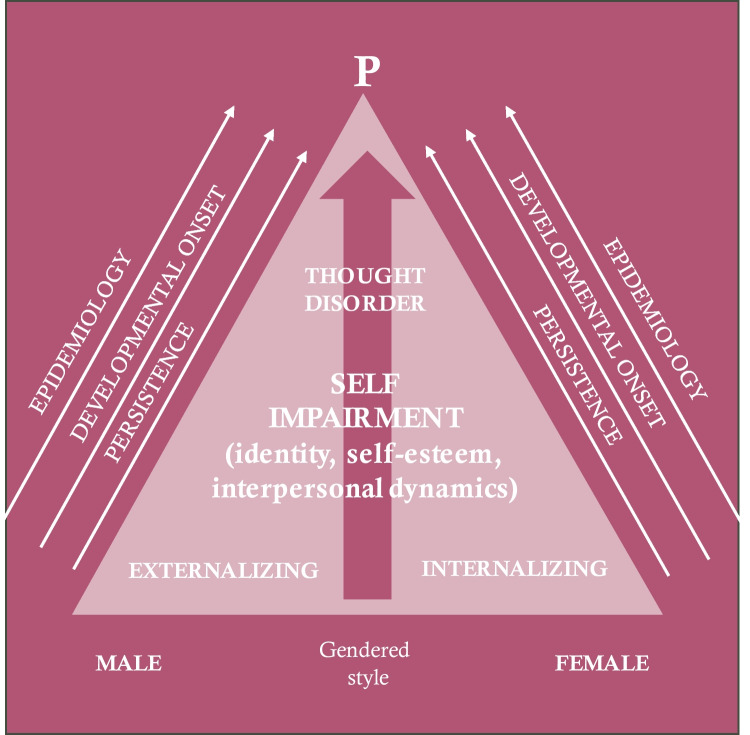
A developmental model for psychopathology. Adapted from Sharp and Wall (2018), a developmental model for psychopathology (p) during adolescence that integrates Criterion A (APA, 2013) conceptualized as self-dysregulation (Sharp, 2020) and considers different self-related aspects that might show more borderline-like (identity integration) or narcissistic-like (self-esteem regulation) presentations

Consistent with a transdiagnostic perspective, including narcissistic features (i.e., imbalances in self-esteem regulation and need for external validation and acknowledgment) can potentially enhance our comprehension of p-factor variance when integrated as a supplementary element alongside borderline personality disorder features. Figure 1, adapted from Caspi et al. (2014) and Sharp and Wall (2018), visually exemplifies the above model. This model integrates impairments in self-regulation as the core of personality pathology, encompassing not only borderline features but also narcissistic features (regulation of self-esteem). Including narcissistic grandiosity and vulnerability can help understand how adolescents’ challenges in self-development - encompassing self-esteem (Ronningstam, 2011) - are associated with the p-factor. Narcissistic grandiosity reflects a defensive expansion of the self and channels interpersonal aggression, whereas narcissistic vulnerability is associated with feelings of insecurity, interpersonal hypersensitivity, and shame (Barry et al., 2019; Chopik & Grimm, 2019; Chrétien et al., 2018; Di Pierro et al., 2017, 2019, 2022; Ensink et al., 2017). Adolescents with grandiose narcissism traits may resort to interpersonal exploitation and aggression to assert their perceived superiority over their peers and bolster their grandiose self-representation (Barry et al., 2003, 2007; Brailovskaia et al., 2020; Kaufman et al., 2020). Adolescents with vulnerable narcissism traits might manifest behavioral difficulties, including aggressive behaviors, lack of empathy, internalizing problems, and addiction (Barry et al., 2007; Bilevicius et al., 2019; Treeby & Bruno, 2012). Considering gender differences, a meta-analytic study on gender differences in narcissism suggests that males might display more grandiose traits than girls. However, no specific gender differences in vulnerability have been identified (Grijalva et al., 2015). However, gender differences in narcissism remain under-researched (Chrétien et al., 2018).

### The Present Study

In summary, research to date suggests that p is a transdiagnostic psychopathology factor accounting for shared variance that traditional models do not capture (Carver et al., 2017; Laceulle et al., 2020; Tackett et al., 2013) and which has been replicated in adolescent populations (Allegrini et al., 2020; Laceulle et al., 2015; Murray et al., 2016; Neumann et al., 2020; Patalay et al., 2015). In addition, recent theoretical models propose that maladaptive personality, conceptualized as impairments in self-development, can be expected to be associated with this general psychopathology factor (Sharp & Wall, 2018) (Fig. 1). However, this requires testing, as research on the association between maladaptive personality and the p-factor remains scarce. The current study aims to address these gaps in research on the p-factor and provide evidence of the specific associations with borderline and narcissistic traits in adolescents. Study 1 aims to test different theoretical models for general adolescent psychopathology (p-factor), aiming at gender invariance. Study 2 aims to explore the associations between borderline and narcissistic traits with general psychopathology, over and beyond their associations with externalizing and internalizing problems, and to examine gender differences in these associations. Based on previous findings (Caspi et al., 2014; Murray et al., 2016), we hypothesized that a bi-factor model of general psychopathology would better fit the data than a first-order or a hierarchical model. Although there are no previous studies examining the p-factor and both borderline personality disorder and narcissistic traits, based on previous contributions (Choate et al., 2023; Sharp & Wall, 2018) focusing on borderline personality disorder features, we hypothesize that borderline and narcissistic traits will display significant associations with the p-factor, over and beyond associations with externalizing and internalizing symptoms. While gender differences have been reported in externalizing and internalizing psychopathology (Sharp et al., 2018), as well as in narcissistic grandiosity (Grijalva et al., 2015; Johnson et al., 2003), no previous studies have examined gender differences in associations between maladaptive personality traits and general psychopathology. For this reason, we have no specific hypotheses regarding gender.

## Study 1 - A Model for Psychopathology in Adolescence

### Method

#### Design and Procedures

We collected cross-sectional data from Italian secondary school students and obtained informed consent from parents and adolescents. Students were assigned a unique reference code to protect their anonymity and provided a private web link to complete self-report questionnaires. The study adhered to APA ethical standards and the Declaration of Helsinki, and the University of Milan-Bicocca Ethical Committee approved all materials and procedures.

#### Participants

To determine the minimum number of participants required to detect at least small effects, an a-priori power analysis was conducted using the R package semPower (Moshagen, 2021). We set Alpha and RMSEA levels to .05. Results indicated that for three latent and eight observed variables, the required sample size to achieve 80% power to reject a wrong model was *N* = 579. Thus, the obtained sample was sufficiently powered.

Participants were 974 cisgender adolescents (63% assigned females at birth; age range: 13—19; M*age* = 16.68, *SD* = 1.40). The majority were Italian natives (*N* = 897; 92.09%). Most adolescents (84.22%) reported living with both parents, and the remainder lived with their mother (13.44%) or father (2.34%). 98% of adolescents reported having at least one significant friend, and 53% said they had three or more friends.

#### Materials

Participants reported internalizing and externalizing problems.

The *Youth Self Report* (YSR) (Achenbach & Rescorla, 2001) is a 112-item self-report measure that assesses general psychological and behavioral difficulties. The Italian translation of the YSR was used for this study (Frigerio et al., 2004). Each item is scored on a 3-point Likert scale (0 = "not true" to 2 = "very or often true"). The measure yields a Total Problems score reflecting general pathological functioning and two comprehensive subscales of Externalizing problems and Internalizing problems, respectively. For this study, we utilized the YSR subscales that include eight dimensions of psychological difficulties: Anxious/Depressed Symptoms, Withdrawn/Depressed Symptoms, Somatic Complaints, Social Problems, Aggressive Behavior, Rule-breaking Behavior, Attention Problems, and Thought Problems. Higher scores indicate higher psychological problems in the specific dimension. All scales showed acceptable to good internal consistency (range α:.73 - .88). To establish p, we used the eight narrowband subscales (Cervin et al., 2021).

#### Statistical Analyses

Statistical analyses were conducted using R ver. 4.3.1 (R Core Team, 2023). In addition, we used descriptive statistics to explore the participants' general characteristics using the *psych* package (Revelle & Revelle, 2015). To test our main hypotheses, we performed structural equation modeling (SEM) using the *lavaan* package (Rosseel et al., 2017).

We adopted a stepwise approach to establishing the best-fitting model for psychopathology that included all YSR subscales. First, we tested a model with latent variables of Internalization and Externalization as correlated first-order factors (Model 1); second, we tested a bi-factor model with a general factor of psychopathology (p-factor) that encompasses latent variables of psychopathology and Internalization and Externalization as orthogonal latent variables of psychopathology (Model 2); third, we tested a hierarchical model with a second-order factor of psychopathology (p-factor) that encompasses latent factors of Internalization and Externalization (Model 3).

In line with Caspi et al. (2014), factor loadings were assessed to highlight the utility of a bi-factor model (Model 2) compared to a correlated factors-only model (Model 1). Indeed, following Demars's suggestions (DeMars, 2013), when testing a bi-factor model, if loadings are low on the specific factors and high on the general factors, only the general factors score carries a reliable interpretation.

We computed all models using a weighted least squares—mean and variance adjusted (WLSMV) estimator to account for Likert-based ordinal measurements (Li, 2016). The fit of the model was evaluated by accounting for complementary goodness of fit indexes (Ullman & Bentler, 2012): chi-square (χ^2^) statistic (if χ^2^ is not significant, it means that model fit with the observed data; however, this statistic is sensitive to sample size and needs interpretation adopting a multifaceted approach; Bollen, 1989); Comparative Fit Index (CFI) and Tucker-Lewis Index (TLI) (values ≥ .95 indicate a good fit, values ≥ .90 indicate an adequate fit); Root Mean Square Error of Approximation (RMSEA) (values < .05 indicate an excellent model fit, values between .05–.08 moderate fit, and values between .08–.10 acceptable fit, such as the non-statistical significance of its associated 90% confidence interval).

To assess gender invariance, we used multi-group SEM accounting for (a) a configural invariance model with invariant factor loading patterns, (b) a metric invariance model with invariant factor loadings, and (c) a scalar invariance model with invariant factor loadings and intercepts; (d) a residual factorial invariance model with invariant indicator residual variances.

As the WLSMV estimator does not produce comparative model fit indices such as Akaike or Bayesian information criterion, we relied on the difference in χ^2^ (△χ^2^) test between nested models to identify the best fitting model.

## Results

**Fig. 2:**
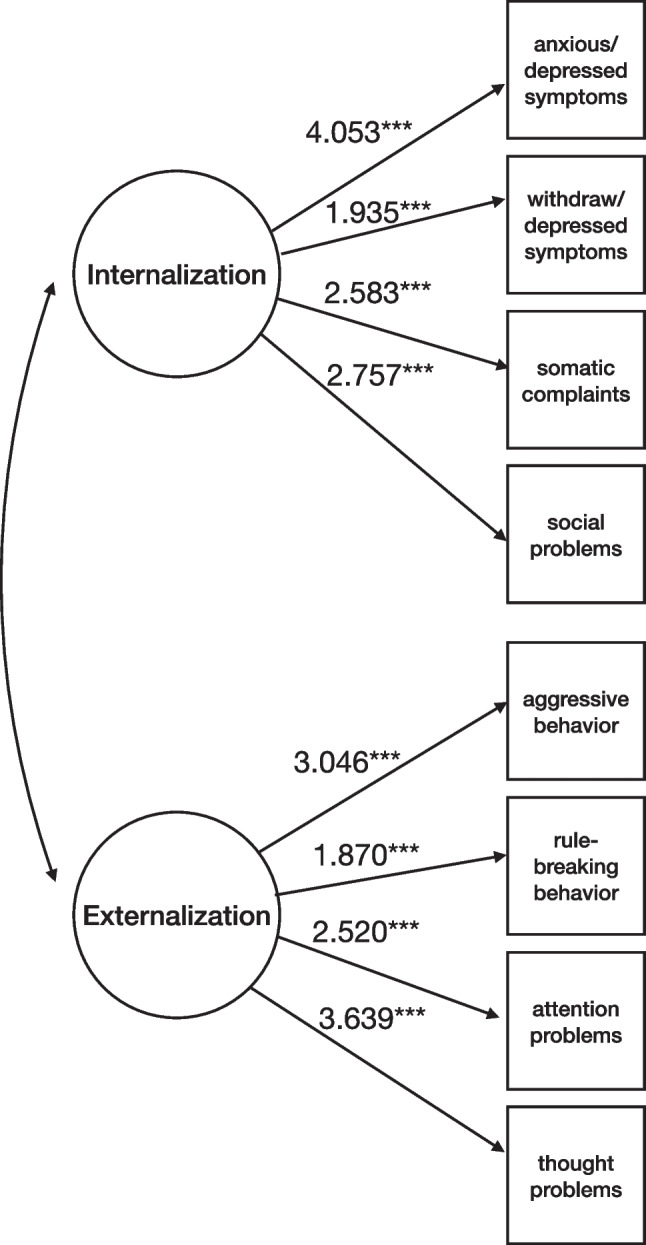
Model 1 for latent variables of psychopathology represented by first-order correlated factors of Internalization and Externalization (N = 974). Note. Solid lines represent statistically significant estimates for latent factors’ indicators, and dashed lines represent nonsignificant ones. Internalization and Externalization = YSR-112 (Achenbach & Rescorla, 2001). ***p ≤ .001; **p ≤ .01; *p ≤ .05

Model 1 included latent variables of psychopathology represented by first-order correlated factors of Internalization and Externalization (Fig. 2). The fit indices of the model showed to be adequate (χ^2^(*df*) = 112.928(19), *p* < .001; χ^2^/ *df* = 5.89; CFI = .969; TLI = .950; RMSEA = .071 [90% CI (.059, .084)], *p* = .003). Standardized loadings were positive: average loadings for Externalization and Internalization were .684 and .748, respectively.

**Fig. 3:**
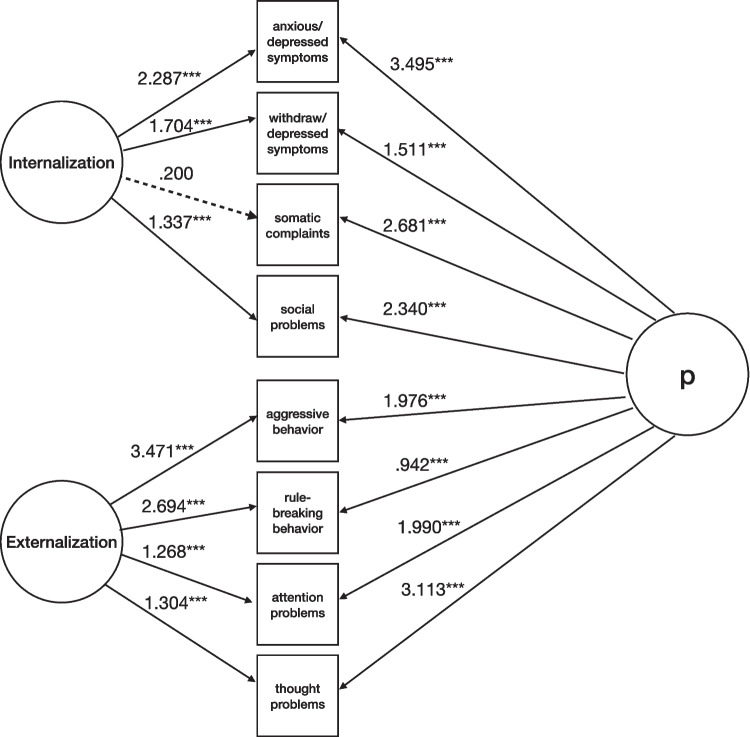
Model 2: bi-factor model with a general factor of psychopathology (p-factor) encompassing latent variables of psychopathology and orthogonal latent variables of Internalization and Externalization (N = 974). Note. Solid lines represent statistically significant standardized estimates for latent factors’ indicators, and dashed lines represent nonsignificant ones. Internalization and Externalization = YSR-112 (Achenbach & Rescorla, 2001). ***p ≤ .001; **p ≤ .01; *p ≤ .05

Model 2 tested a bi-factor model with a general factor of psychopathology (p-factor) encompassing latent variables of psychopathology and orthogonal latent variables of Internalization and Externalization (Fig. 3). The fit indices suggested the model to fit adequately (χ^2^(*df*) = 23.545(12), *p* = .201; χ^2^/*df* = 1.96; CFI = .996; TLI = .988; RMSEA = .031 [90% CI (.011, .050)], *p* = .948). Standardized loadings were positive: average loadings for the p-factor were .578, while average loadings for Externalization and Internalization were .534 and .367, respectively.

Considering loadings on Externalization: Thought Problems were .822 in Model 1 and .295 in Model 2, while the loading on p-factor was .703; Aggressive Behavior was .673 in Model 1 and .767 in Model 2, while the loading on p-factor was .437; Rule-breaking Behavior was .472 in Model 1 and .679 in Model 2, while the loading on p-factor was .437; Attention Problems were .769 in Model 1 and .382 in Model 2, while the loading on p-factor was .607.

Considering loadings on Internalization: Social Problems were .846 in Model 1 and .410 in Model 2, while the loading on p-factor was .718; Anxious/Depressed Symptoms were .799 in Model 1 and .451 in Model 2, while the loading on p-factor was .689; Withdrawn/Depressed Symptoms were .626 in Model 1 and .551 in Model 2, while the loading on p-factor was .488; Somatic Complaints were .722 in Model 1 and .056 in Model 2, while the loading on p-factor was .750.

Thus, data indicate that most of the propensity to Thought Problems, Attention Problems, Somatic Complaints, and Social Problems indicates general psychopathology instead of Externalization/Internalization. Instead, the propensity to Aggressive Behavior, Rule-breaking Behavior, Anxious/Depressed Symptoms, and Withdrawn/Depressed Symptoms stem from the combination of Externalization/Internalization and general psychopathology.

Model 3 included a hierarchical model with a second-order factor of psychopathology (p-factor) encompassing Internalization and Externalization latent factors. The model did not converge; hence, it was discarded.

χ^2^ difference showed that Model 2 fitted the data significantly better than Model 1 (Δχ^2^ = 89.384; *p* < .001).

When testing for gender invariance of Model 2, the baseline model showed an acceptable fit, supporting configural invariance. In the next step, equality constraints were imposed on all factor loadings to examine metric invariance. The resulting model also achieved an acceptable fit (i.e., items were related to the latent factor equivalently across groups) (χ^2^ (df) = 136.687 (42), *p* = .001; χ^2^/df = 1.12; CFI = .971; TLI = .953; RMSEA = .068 [90% CI (.056, .081)], *p* = .010). Equality constraints were imposed on all thresholds to test scalar invariance; this model did not achieve an acceptable fit.

## Study 2 - Associations between Borderline and Narcissistic Personality Traits and Psychopathology

### Method

#### Study Design and Procedures

We used the same study design and procedure as in Study 1 for the current study.

#### Participants

To determine the minimum number of participants required to detect at least small effects, an a-priori power analysis was conducted using the R package semPower (Moshagen, 2021). Alpha and RMSEA levels were set to .05. Results indicated that for three latent and 11 observed variables, the required sample size to achieve 80% power to reject a wrong model was *N* = 317. Thus, the obtained sample is sufficiently powered.

Participants were 725 cisgender adolescents (64.5% assigned females at birth; age range: 13–19; *M* age = 16.22, *SD* = 1.32). All participants were fluent in Italian; most were Italian natives (*N* = 665; 91.7%). Most adolescents (80.55%) reported living with both parents, 15.72% lived with their mother, 2.02% with their father, and 1% with other relatives. Most adolescents (97%) reported having at least one significant friend, with 48% reporting three or more.

#### Materials

Participants reported internalizing and externalizing problems (YSR) (Achenbach & Rescorla, 2001), borderline personality traits, and narcissistic personality traits.

The *Borderline Personality Feature Scale for Children – 11* (BPFSC-11) (Sharp et al., 2014) is an 11-item measure assessing borderline personality traits in children and adolescents aged 9 to 18. The Italian translation of the BPFSC-11 was used for this study (Fossati et al., 2016). Items are rated on a 5-point Likert scale (1 = "not true at all"; 5 = "always true"), describing borderline personality traits such as identity problems, negative interpersonal relationships, and affective instability. The BPFSC-11 yields a total score (11–55), measuring the overall borderline personality features (BPF) level. Higher scores indicate higher borderline personality traits. The scale showed adequate internal consistency (α = .77).

The *Pathological Narcissism Inventory* (PNI) (Pincus et al., 2009) is a 52-item self-report questionnaire assessing narcissistic personality traits along two different dimensions: Narcissistic Vulnerability (NV) and Narcissistic Grandiosity (NG). The Italian translation of the PNI was used for this study (Somma et al., 2020). NV includes features of Contingent Self-Esteem (fluctuations in self-esteem levels in the absence of external sources of admiration and recognition), Hiding the Self (unwillingness to show others one’s faults and needs), Devaluing (disinterest in others who do not provide admiration, as well as shame over needing recognition from disappointing others), and Entitlement Rage (proneness to experience anger when expectations are not met). NG encompasses Exploitativeness (a manipulative interpersonal orientation), Self-Sacrificing Self-Enhancement (the use of purportedly altruistic acts to sustain an inflated self-image), and Grandiose Fantasies (engagement in compensatory fantasies of gaining success, recognition, and admiration). Higher scores indicate higher narcissistic traits. Both scales showed good internal consistency (NG: α = .83; NV: α = .90).

#### Statistical Analyses

Statistical analyses were conducted using R ver. 4.3.1 (R Core Team, 2023). In addition, we used descriptive statistics to explore the participants' general characteristics using the *psych* package (Revelle & Revelle, 2015). To test our main hypotheses, we performed structural equation modeling (SEM) using the *lavaan* package (Rosseel et al., 2017).

We used the same statistical procedure and indicators as in Study 1 to explore the contribution to the p-factor of borderline and narcissistic personality traits, over and beyond their associations with externalizing and internalizing symptoms. To assess gender differences, we used multi-group SEM with no equality constraints to highlight gender-specific associations between BPF, NG, NV, and psychopathology.

The models' explanatory powers were assessed with path coefficients and *R*^*2*^.

#### Results

**Fig. 4:**
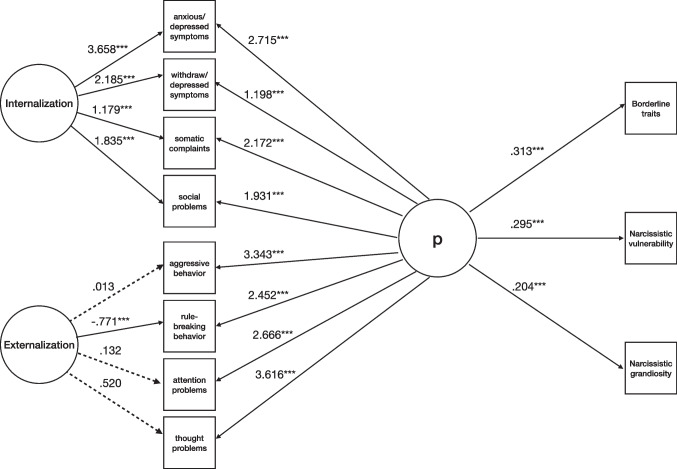
Model for the associations between borderline and narcissistic personality traits and psychopathology (N = 725). Note. Solid lines represent statistically significant estimates for latent factors’ indicators, and dashed lines represent nonsignificant ones. For clarity, estimates from Borderline Features, Narcissistic Vulnerability, and Narcissistic Grandiosity to latent factors of Internalization and Externalization were not included in the Figure. Internalization and Externalization = YSR-112 (Achenbach & Rescorla, 2001); Borderline Traits = BPFSC-11 (Sharp et al., 2014); Narcissistic Vulnerability and Narcissistic Grandiosity = PNI (Pincus et al., 2009). ***p ≤ .001; **p ≤ .01; *p ≤ .05

First, we included maladaptive personality traits (borderline and narcissistic ones) as indicators of psychopathology (Fig. 4). The fit indices showed adequate model fit (χ^2^(*df*) = 68.153(30), *p* < .001; χ^2^/*df* = 2.271; CFI = .990; TLI = .982; RMSEA = .044 [90% CI (.038, .058)], *p* = .747). For the p-factor, standardized loadings were positive and averaged .549 (see Supplementary Table 1 for detailed loadings).

After considering Externalization and Internalization, BPF, NV, and NG contributed significantly to general psychopathology. The model explained a total variance of 90% of NV, 59% of BPF, and 47% of NG.

Second, we explored gender differences in the associations between maladaptive personality traits and psychopathology. The fit indices were indicative of adequate model fit (χ^2^(*df*) = 65.004 (60), *p* = .307; χ^2^/*df* = 1.083; CFI = .998; TLI = .994; RMSEA = .016 [90% CI (.000, .038)], p = .998).

**Fig. 5:**
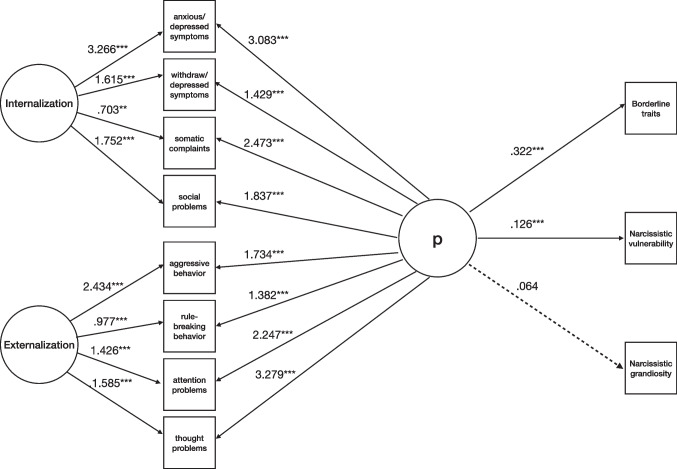
Model for the associations between borderline and narcissistic personality traits and psychopathology in females (N = 465). Note. Solid lines represent statistically significant estimates for latent factors’ indicators, and dashed lines represent nonsignificant ones. For clarity, estimates from Borderline Features, Narcissistic Vulnerability, and Narcissistic Grandiosity to latent factors of Internalization and Externalization were not included in the Figure. Internalization and Externalization = YSR-112 (Achenbach & Rescorla, 2001); Borderline Traits = BPFSC-11 (Sharp et al., 2014); Narcissistic Vulnerability and Narcissistic Grandiosity = PNI (Pincus et al., 2009). ***p ≤ .001; **p ≤ .01; *p ≤ .05

After considering Externalization and Internalization in females, BPF and NV contributed significantly to general psychopathology but not NG (Fig. 5). For the p-factor, standardized loadings were positive and averaged .479 (see Supplementary Table 2 for detailed loadings). The model explained a total variance of 94% of NV, 64% of BPF, and 33% of NG.

**Fig. 6:**
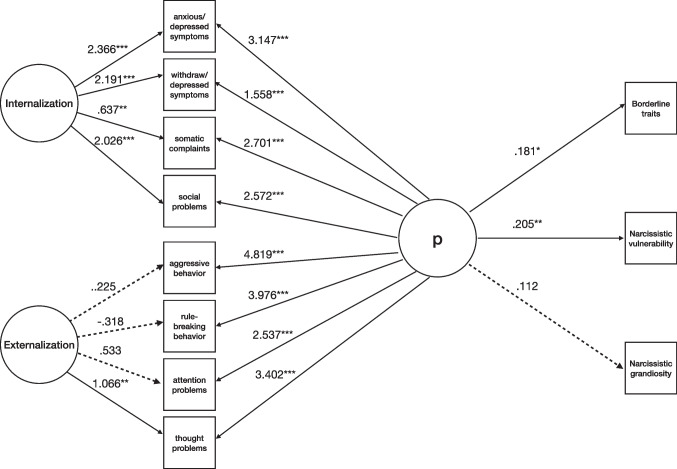
Model for the associations between borderline and narcissistic personality traits and psychopathology in males (N = 260). Note. Solid lines represent statistically significant estimates for latent factors’ indicators, and dashed lines represent nonsignificant ones. For clarity, estimates from Borderline Features, Narcissistic Vulnerability, and Narcissistic Grandiosity to latent factors of Internalization and Externalization were not included in the Figure. Internalization and Externalization = YSR-112 (Achenbach & Rescorla, 2001); Borderline Traits = BPFSC-11 (Sharp et al., 2014); Narcissistic Vulnerability and Narcissistic Grandiosity = PNI (Pincus et al., 2009). ***p ≤ .001; **p ≤ .01; *p ≤ .05

After considering Externalization and Internalization in males, BPF and NV contributed significantly to general psychopathology but not NG (Fig. 6). For the p-factor, standardized loadings were positive and averaged .577 (see Supplementary Table 3 for detailed loadings). The model explained a total variance of 89% of NV, 60% of NG, and 52% of BPF.

#### General Discussion

This study aimed to examine the p-factor in adolescence and clarify the contribution of borderline and narcissistic traits to general psychopathology in females and males.

#### Is a Bi-factor Model for General Psychopathology Useful in Adolescence?

Consistent with our initial hypothesis, the findings of Study 1 confirm the superiority of the bi-factor model compared to a solely correlated-factors model (DeMars, 2013).

Moreover, the findings indicate that the p-factor highlights specific aspects of externalization and internalization. Specifically, accounting for the p-factor showed that emotional or behavioral problems did not encompass Thought Problems. This is consistent with previous research suggesting that Thought Problems may be a supplementary category in bi-factor models in addition to behavioral dysregulation and anxiety/depression/somatic aspects (Caspi et al., 2014; Haltigan et al., 2018). Furthermore, the findings suggest that the externalizing dimension primarily incorporates behavioral dysregulation associated with aggression (e.g., Aggressive Behavior and Rule-breaking Behavior) rather than dysregulation linked to inattention or hyperactivity (e.g., Attention Problems). This finding is interesting as it suggests that inattention/hyperactivity may be better explained by general psychopathology than externalizing. Additionally, while anxious and depressive characteristics contributed to internalization, the findings indicate that both interpersonal challenges (e.g., Social Problems) and somatic manifestations (e.g., Somatic Complaints) are more adequately accounted for by general psychopathology. Indeed, this aligns with the findings from another recent study (Marek et al., 2020), which showed that somatization represents a distinct factor separate from internalization and externalization.

While our findings provide further evidence of the utility of examining general liability to psychopathology, the interpretation of the p-factor, above being a shared variance that other factors do not capture, remains a topic of debate. Some have cautioned that it is “important not to *reify* the general factor” (Laceulle et al., 2015, p. 858). For example, the p-factor might grasp commonality in the variance of all psychopathology or reflect distress or emotional responsivity (Carver et al., 2017). Alternatively, it might also describe a dynamic process in which a network of dimensions interacts (McElroy et al., 2018).

#### Is Self-dysregulation Contributing to General Psychopathology?

In line with our initial hypothesis, the study's primary finding is that impairments in self-functioning, as evident in personality difficulties, contributed to general psychopathology captured with the p-factor. Indeed, borderline traits were positively associated with general psychopathology, indicating that higher levels of these traits correspond to a higher p-factor. This finding aligns with the literature that suggests that the core of personality pathology is adequately described by identity instability, interpersonal problems, and emotion dysregulation (Bender et al., 2011; Morey et al., 2011; Sharp & Fonagy, 2015; Sharp et al., 2018). Similarly, narcissistic features were positively associated with the p-factor, suggesting that self-dysregulation, as manifested in self-esteem regulation imbalances, is linked to general psychopathology (Ronningstam, 2011). However, when looking at factor loadings, the findings suggest that the contribution of narcissistic grandiosity is also associated with externalizing manifestations. This association is further clarified when considering gender differences. Indeed, in female and male adolescents, borderline features and narcissistic vulnerability, but not narcissistic grandiosity, contributed to general psychopathology.

This finding opens a reflection on the development of the self as a transdiagnostic dimension entailing adolescents’ self-image and self-esteem concerning themselves and their interpersonal world. Indeed, developing a coherent self is a crucial task of this developmental phase (Ensink et al., 2015; Sharp, 2020; Normandin et al., 2023; Fontana et al., 2021), and dysregulation in self-development might prove pivotal in shaping adolescents’ liability to psychopathology. In particular, borderline features might express identity confusion, emotional dysregulation, and interpersonal problems, while narcissistic vulnerability might capture adolescents’ fluctuations in self-esteem and interpersonal sensitivity (Benzi et al., 2023b; Diamond et al., 2021; Ronningstam, 2011).

Moreover, considering that we used data obtained from the self-reports of adolescents, we can also see the findings as suggesting that self-dysregulation might impact how intensely adolescents perceive psychopathology as expressed by p (Haltigan et al., 2018; Laceulle et al., 2020) and speculate that the higher this intensity (i.e., feelings of identity confusion, feelings of self-vulnerability) the higher the impact on adolescents’ interpersonal functioning.

#### Unpacking the p-factor: Limitations and Considerations for Future Research

While the study had several strengths, including using a large sample of adolescents, the study also has certain limitations. First, our exploration of the p-factor is limited as only a selection of psychopathology symptoms was included. Further studies should account for a broader range of symptomatology that might especially be relevant during adolescence (i.e., autism spectrum). Second, our study utilized cross-sectional data, which limits our ability to draw causal inferences and only allows for identifying associations. Third, latent variables were explored with sum scores and not at the item level. Fourth, we only employed self-report measures in our study. Despite the advantage of quicker data collection during school hours (as in the case of our research), using only self-reports might introduce biases (i.e., social desirability). Thus, future research should adopt a multi-informant perspective (i.e., including parents and teachers) to provide a more comprehensive view of adolescents’ functioning. Future research would benefit from incorporating semi-structured interviews to assess psychopathology more accurately. Fifth, despite the importance of exploring psychopathology and sub-threshold psychopathology in community samples, it is important to replicate our study in larger and culturally diverse adolescent samples and clinical populations. Sixth, in our data collection, we did not gather information on adolescents’ family income: this is an important variable that should be considered in future studies as it might contribute to a better understanding of adolescents’ level of functioning and general psychological distress. Seventh, a significant challenge associated with the bifactor modeling approach is its inherent flexibility. Indeed, while it enables a comprehensive understanding of the data by capturing both general and specific sources of variance, it can also lead to potential overfitting, providing a misleading impression of its superiority (Lahey et al., 2017). Future studies might benefit from cross-validating bifactor results with other modeling strategies to ensure robustness and avoid such pitfalls. Eight, although our sample size provided adequate statistical power for the total sample, we did not explore (i.e., simulation models) the adequate power for SEM multi-group models such as the ones we have tested. Ongoing data collection will provide larger samples to replicate our study and ensure the generalizability of our findings. Finally, borderline and narcissistic features were used as proxies to explore the level of personality functioning as conceptualized according to a dimensional model of emerging personality (Benzi et al., 2023a, b; Ensink et al., 2015; Sharp et al., 2018). Future studies should use specific levels of personality functioning measures to test relevant theoretical models more closely.

In conclusion, our findings provide new evidence that the developmental trajectory of the p-factor encompasses not only internalizing and externalizing difficulties but also emerging personality pathology. Consistent with a transdiagnostic perspective, including borderline personality and narcissistic features, enhances our comprehension of the p-factor. This model integrates impairments in self-development, specifically in self-regulation (borderline features) and self-esteem (narcissistic features), at the core of personality pathology and liability to psychopathology.

### Supplementary Information

Below is the link to the electronic supplementary material.

Supplementary file1 (DOCX 41 KB)

## Data Availability

The data that support the findings of this study are not openly available due to reasons of sensitivity and are available from the corresponding author upon reasonable request.
