# Supplementary material for: Unpacking the p-factor. Associations Between Maladaptive Personality Traits and General Psychopathology in Female and Male Adolescents
Source: Res Child Adolesc Psychopathol. 2023 Nov 8;52(3):473–86. doi: 10.1007/s10802-023-01146-w (PMC10896943; doi:10.1007/s10802-023-01146-w)
Supplement: Supplementary file 1 — Supplementary file1 (DOCX 41 KB) [file 10802_2023_1146_MOESM1_ESM.docx]

**Supplementary Table 1. Model for the associations between borderline and narcissistic personality traits with latent factors of psychopathology (p), Internalization, and Externalization**

|  |  |  |  |  | **95% Confidence Interval** | |  |
| --- | --- | --- | --- | --- | --- | --- | --- |
| **Latent variable** | **Indicator** | **Estimate** | **SE** | **z-value** | **Lower** | **Upper** | **Standardized loadings** |
| Externalization | Aggressive Behavior | .013 | .282 | .045 | -.540 | .565 | .003 |
|  | Rule-breaking Behavior | -.771*** | .212 | -3.643 | -1.186 | -.356 | -.179 |
|  | Attention Problems | .132 | .241 | .548 | -.340 | .604 | .039 |
|  | Thought Problems | .520 | .317 | 1.637 | -.102 | 1.142 | .114 |
|  | Borderline Features | .296*** | .035 | 8.360 | .226 | .365 | .457 |
|  | Narcissistic Vulnerability | .564*** | .030 | 18.867 | .505 | .622 | .767 |
|  | Narcissistic Grandiosity | .412*** | .036 | 11.446 | .341 | .482 | .607 |
| Internalization | Anxious/Depressed Symptoms | 3.658*** | .186 | 19.703 | 3.294 | 4.022 | .702 |
|  | Withdrawn/Depressed Symptoms | 2.185*** | .126 | 17.303 | 1.938 | 2.433 | .650 |
|  | Somatic Complaints | 1.179*** | .145 | 8.108 | .894 | 1.464 | .313 |
|  | Social Problems | 1.835*** | .116 | 15.866 | 1.608 | 2.061 | .537 |
|  | Borderline Features | .248*** | .025 | 9.802 | .199 | .298 | .384 |
|  | Narcissistic Vulnerability | .288*** | .040 | 7.215 | .210 | .366 | .391 |
|  | Narcissistic Grandiosity | -.068* | .033 | -2.046 | -.132 | -.003 | -.100 |
| p | Anxious/Depressed Symptoms | 2.715*** | .226 | 12.012 | 2.272 | 3.159 | .521 |
|  | Withdrawn/Depressed Symptoms | 1.198*** | .151 | 7.946 | .903 | 1.494 | .356 |
|  | Somatic Complaints | 2.172*** | .189 | 11.465 | 1.801 | 2.544 | .576 |
|  | Social Problems | 1.931*** | .177 | 1.898 | 1.584 | 2.278 | .565 |
|  | Aggressive Behavior | 3.343*** | .240 | 13.911 | 2.872 | 3.814 | .684 |
|  | Rule-breaking Behavior | 2.452*** | .269 | 9.117 | 1.925 | 2.980 | .569 |
|  | Attention Problems | 2.666*** | .129 | 2.636 | 2.413 | 2.920 | .783 |
|  | Thought Problems | 3.616*** | .194 | 18.648 | 3.236 | 3.996 | .795 |
|  | Borderline Features | .313*** | .047 | 6.597 | .220 | .406 | .484 |
|  | Narcissistic Vulnerability | .295*** | .055 | 5.417 | .189 | .402 | .402 |
|  | Narcissistic Grandiosity | .204*** | .047 | 4.323 | .111 | .296 | .301 |

Anxious/Depressed Symptoms, Withdrawn/Depressed Symptoms, Somatic Complaints, Social Problems, Aggressive Behavior, Rule-breaking Behavior, Attention Problems, Thought Problems= YSR-112 (Achenbach & Rescorla, 2001); Borderline Features = BPFSC-11 (Sharp et al., 2014); Narcissistic Vulnerability and Narcissistic Grandiosity = PNI (Pincus et al., 2009). *** *p*≤.001; ***p*≤.01; **p*≤.05

**Supplementary Table 2. Model for the associations between borderline and narcissistic personality traits with latent factors of psychopathology (p), Internalization, and Externalization in females**

|  |  |  |  |  | **95% Confidence Interval** | |  |
| --- | --- | --- | --- | --- | --- | --- | --- |
| **Latent variable** | **Indicator** | **Estimate** | **SE** | **z-value** | **Lower** | **Upper** | **Standardized loadings** |
| Externalization | Aggressive Behavior | 2.434*** | .281 | 8.671 | 1.884 | 2.984 | .563 |
|  | Rule-breaking Behavior | .977*** | .229 | 4.265 | .528 | 1.426 | .287 |
|  | Attention Problems | 1.426*** | .215 | 6.630 | 1.004 | 1.847 | .427 |
|  | Thought Problems | 1.585*** | .286 | 5.546 | 1.025 | 2.146 | .348 |
|  | Borderline Features | .258*** | .026 | 1.080 | .208 | .308 | .410 |
|  | Narcissistic Vulnerability | .446*** | .054 | 8.208 | .340 | .553 | .606 |
|  | Narcissistic Grandiosity | .328*** | .033 | 9.901 | .263 | .393 | .541 |
| Internalization | Anxious/Depressed Symptoms | 3.266*** | .283 | 11.543 | 2.711 | 3.820 | .633 |
|  | Withdrawn/Depressed Symptoms | 1.615*** | .177 | 9.127 | 1.268 | 1.962 | .484 |
|  | Somatic Complaints | .703** | .222 | 3.174 | .269 | 1.138 | .192 |
|  | Social Problems | 1.752*** | .169 | 1.348 | 1.420 | 2.084 | .558 |
|  | Borderline Features | .287*** | .028 | 1.323 | .233 | .342 | .457 |
|  | Narcissistic Vulnerability | .544*** | .050 | 1.965 | .447 | .642 | .739 |
|  | Narcissistic Grandiosity | .089* | .039 | 2.251 | .011 | .166 | .146 |
| p | Anxious/Depressed Symptoms | 3.083*** | .327 | 9.420 | 2.442 | 3.725 | .597 |
|  | Withdrawn/Depressed Symptoms | 1.429*** | .201 | 7.125 | 1.036 | 1.822 | .428 |
|  | Somatic Complaints | 2.473*** | .209 | 11.825 | 2.063 | 2.883 | .673 |
|  | Social Problems | 1.837*** | .214 | 8.591 | 1.418 | 2.256 | .585 |
|  | Aggressive Behavior | 1.734*** | .281 | 6.174 | 1.184 | 2.285 | .401 |
|  | Rule-breaking Behavior | 1.382*** | .227 | 6.077 | .936 | 1.828 | .407 |
|  | Attention Problems | 2.247*** | .191 | 11.773 | 1.873 | 2.622 | .673 |
|  | Thought Problems | 3.279*** | .281 | 11.656 | 2.727 | 3.830 | .719 |
|  | Borderline Features | .322*** | .034 | 9.447 | .255 | .389 | .511 |
|  | Narcissistic Vulnerability | .126** | .045 | 2.780 | .037 | .215 | .171 |
|  | Narcissistic Grandiosity | .064 | .039 | 1.629 | -.013 | .140 | .105 |

Anxious/Depressed Symptoms, Withdrawn/Depressed Symptoms, Somatic Complaints, Social Problems, Aggressive Behavior, Rule-breaking Behavior, Attention Problems, Thought Problems= YSR-112 (Achenbach & Rescorla, 2001); Borderline Features = BPFSC-11 (Sharp et al., 2014); Narcissistic Vulnerability and Narcissistic Grandiosity = PNI (Pincus et al., 2009). *** *p*≤.001; ***p*≤.01; **p*≤.05

**Supplementary Table 3. Model for the associations between borderline and narcissistic personality traits with latent factors of psychopathology (p), Internalization, and Externalization in males**

|  |  |  |  |  | **95% Confidence Interval** | |  |
| --- | --- | --- | --- | --- | --- | --- | --- |
| **Latent variable** | **Indicator** | **Estimate** | **SE** | **z-value** | **Lower** | **Upper** | **Standardized loadings** |
| Externalization | Aggressive Behavior | .225 | .488 | .461 | -.732 | 1.182 | .039 |
|  | Rule-breaking Behavior | -.318 | .394 | -.806 | -1.091 | .455 | -.063 |
|  | Attention Problems | .533 | .334 | 1.598 | -.121 | 1.187 | .151 |
|  | Thought Problems | 1.066** | .386 | 2.763 | .310 | 1.822 | .237 |
|  | Borderline Features | .364*** | .043 | 8.521 | .280 | .448 | .578 |
|  | Narcissistic Vulnerability | .590*** | .043 | 13.810 | .506 | .674 | .844 |
|  | Narcissistic Grandiosity | .598*** | .058 | 1.403 | .486 | .711 | .754 |
| Internalization | Anxious/Depressed Symptoms | 2.366*** | .271 | 8.726 | 1.834 | 2.897 | .515 |
|  | Withdrawn/Depressed Symptoms | 2.191*** | .219 | 1.005 | 1.762 | 2.620 | .649 |
|  | Somatic Complaints | .637** | .228 | 2.791 | .190 | 1.085 | .167 |
|  | Social Problems | 2.026*** | .201 | 1.072 | 1.632 | 2.420 | .520 |
|  | Borderline Features | .203*** | .052 | 3.928 | .102 | .304 | .323 |
|  | Narcissistic Vulnerability | .212*** | .062 | 3.437 | .091 | .334 | .304 |
|  | Narcissistic Grandiosity | -.086 | .076 | -1.120 | -.235 | .064 | -.108 |
| p | Anxious/Depressed Symptoms | 3.147*** | .387 | 8.124 | 2.388 | 3.906 | .686 |
|  | Withdrawn/Depressed Symptoms | 1.558*** | .269 | 5.800 | 1.031 | 2.084 | .461 |
|  | Somatic Complaints | 2.701*** | .350 | 7.726 | 2.016 | 3.387 | .710 |
|  | Social Problems | 2.572*** | .341 | 7.539 | 1.904 | 3.241 | .660 |
|  | Aggressive Behavior | 4.819*** | .392 | 12.293 | 4.050 | 5.587 | .845 |
|  | Rule-breaking Behavior | 3.976*** | .406 | 9.800 | 3.181 | 4.771 | .783 |
|  | Attention Problems | 2.537*** | .249 | 1.190 | 2.049 | 3.024 | .720 |
|  | Thought Problems | 3.402*** | .345 | 9.853 | 2.725 | 4.079 | .757 |
|  | Borderline Features | .181* | .076 | 2.372 | .031 | .330 | .287 |
|  | Narcissistic Vulnerability | .205** | .076 | 2.707 | .056 | .353 | .292 |
|  | Narcissistic Grandiosity | .112 | .085 | 1.315 | -.055 | .279 | .141 |

Anxious/Depressed Symptoms, Withdrawn/Depressed Symptoms, Somatic Complaints, Social Problems, Aggressive Behavior, Rule-breaking Behavior, Attention Problems, Thought Problems= YSR-112 (Achenbach & Rescorla, 2001); Borderline Features = BPFSC-11 (Sharp et al., 2014); Narcissistic Vulnerability and Narcissistic Grandiosity = PNI (Pincus et al., 2009). *** *p*≤.001; ***p*≤.01; **p*≤.05
